# Ion concentration polarization (ICP) of proteins at silicon micropillar nanogaps

**DOI:** 10.1371/journal.pone.0223732

**Published:** 2019-11-04

**Authors:** Bochao Lu, Michel M. Maharbiz

**Affiliations:** 1 UC Berkeley-UCSF Graduate Program in Bioengineering, University of California–Berkeley, Berkeley, California, United States of America; 2 Electrical Engineering and Computer Science Department, University of California–Berkeley, Berkeley, California, United States of America; 3 Chan Zuckerberg Biohub, San Francisco, California, United States of America; Universita degli Studi di Pisa, ITALY

## Abstract

Fast detection of low-abundance protein remains a challenge because detection speed is limited by analyte transport to the detection site of a biosensor. In this paper, we demonstrate a scalable fabrication process for producing vertical nanogaps between micropillars which enable ion concentration polarization (ICP) enrichment for fast analyte detection. Compared to horizontal nanochannels, massively paralleled vertical nanogaps not only provide comparable electrokinetics, but also significantly reduce fluid resistance, enabling microbead-based assays. The channels on the device are straightforward to fabricate and scalable using conventional lithography tools. The device is capable of enriching protein molecules by >1000 fold in 10 min. We demonstrate fast detection of IL6 down to 7.4 pg/ml with only a 10 min enrichment period followed by a 5 min incubation. This is a 162-fold enhancement in sensitivity compared to that without enrichment. Our results demonstrate the possibility of using silicon/silica based vertical nanogaps to mimic the function of polymer membranes for the purpose of protein enrichment.

## Introduction

A number of microfluidics based immunoassays have been developed specifically for low abundance target molecules[1], including cantilever-based biosensors[2], surface plasmon resonance (SPR)[3], and nanowire-based immunoassays[4]. However, immunoassays for low concentration proteins remain a challenge because most existing technologies are sensitive to antibody quality and require relatively long incubation times. The sensitivity of most biosensors depends on the affinity of the capture antibody, implying that high sensitivity biosensors require high quality antibodies with a very low dissociation constant, K_d_. In addition, antibody-antigen systems require relatively long incubation times to achieve binding equilibrium. This is especially pronounced at low antigen concentrations [5], particularly at concentrations below the antibody dissociation constant as analyte transport to the biosensor becomes the rate limiting step [6–9]. As a result, incubation times ranging from several hours to overnight are usually necessary for low concentration detection [10]. To overcome these limitations, many sample enrichment technologies have been developed in recent years; these include nano-dispensing [11], size exclusion [12,13], isoelectric focusing [14–17], isotachophoresis [18], amplification stacking [19–22], and dielectrophoresis [23–26]. However, the application of these enrichment methods to immunoassays remains a challenge. Among the difficulties in applying these to immunoassays are the fact that size exclusion depends on the molecule size; sample stacking and isotachophoresis requires special buffer arrangements; and dielectrophoresis requires an expensive, high quality electronic amplifier with high slew rate, low noise, and high power.

In this context, ion concentration polarization (ICP) has captured tremendous interest in the field of electrokinetic preconcentration of biomolecules [27,28]. ICP-based preconcentrators do not require special buffers and reagents, in contrast to sample stacking and isotachophoresis. ICP methods accumulate biomolecules at the boundary of a depletion zone, regardless of the molecule size [29–32]. Since ICP-based devices only require DC voltages, they are much simpler and cheaper than those employing dielectrophoresis [23–26]. Because ICP is a phenomenon that exists at the interface between nanochannels and microchannels, ICP-based protein enrichment was originally achieved on silicon or glass substrates using thin horizontal nanochannels [5,33–37]. Polydimethylsiloxane (PDMS) and Nafion have also been used for nanofluidic preconcentration devices because the Nafion membrane provides similar permselectivity to silica nanochannels and PDMS is a well-known material for prototyping microfluidics [31,32,38–45]. For example, the simplest preconcentrator employs a Nafion membrane deposited in the center of a straight PDMS microchannel [40]. The high difference in conductivity between the Nafion membranes and the PDMS microchannel induce ICP, enriching biomolecules at the anodic side of the Nafion film. However, this simple design is hard to replicate on silicon or silica substrates with horizontal nanochannels, since the horizontal nanochannels alone cannot provide sufficient volumetric flow for immunoassay operations such as sample loading and washing. Thus, in most silicon/silica preconcentrators the horizontal nanochannels must be perpendicular to larger microchannels [5,33,36,37], which results in a more complex structure. Importantly, while PDMS/Nafion devices are excellent for prototyping (i.e. production in the tens of devices per day), silicon/silica-based devices are easier to scale up to high volumes (i.e. tens of thousands of devices per day) given modern photolithography tools and processes.

**Fig 1:**
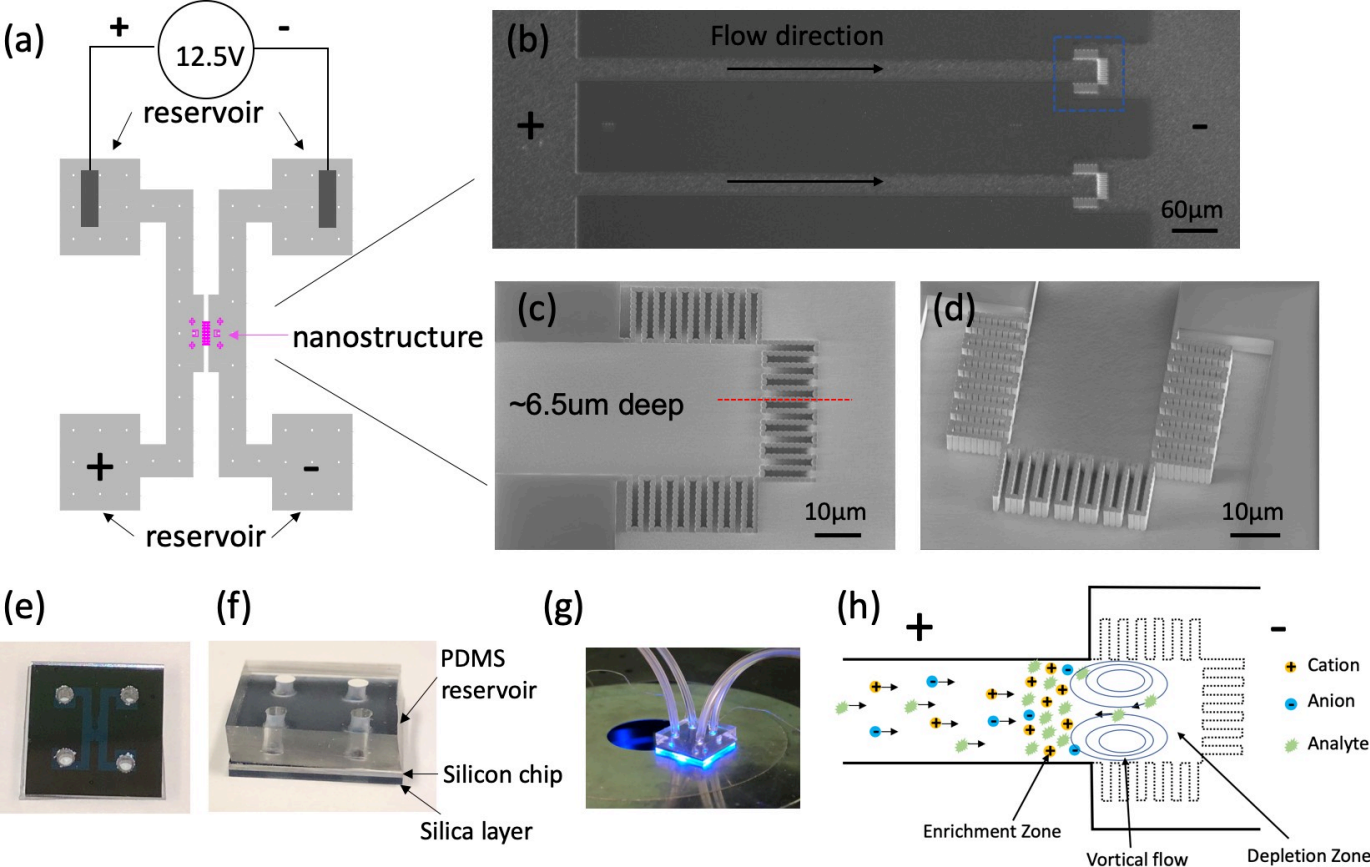
Overview of the preconcentration device. (a) Schematic diagram of the entire device. Two main channels (each with two reservoirs) are connected by the microchannels with nanogaps in the middle. The reservoirs are used to connect external fluidic tubing with the microchannels and to provide contact with electrodes. (b) Close-up of the microchannels connecting the two main channels. These microchannels are 700 μm long, 30 μm wide, 6.5 μm tall, and contain 397 micropillars at one end. Nanogaps between micropillars are approximately 30 nm wide and induce ICP to enrich target proteins. (c) Top view. (d) 30° off-axis. SEM of the nanostructures in the blue box in (b). There are in total 397 micropillars (1um x 1 um x 6.5 um) forming 398 nanogaps. (e)-(g) Pictures of finished chips. Each chip has three layers, PDMS layer for tube connection, silicon layers with all the micro/nano structures, and silica layer for sealing purpose. (h) Schematic of ICP and protein enrichment in front of nanostructures with the anode on the left side.

In this paper, we demonstrate a silicon/silica based preconcentration device with 397 micropillars forming 398 vertical nanogaps between them (Fig 1). The presented process has specific advantages when compared to previous silicon/silica nanochannel designs which require horizontal nanochannels as well as polymer membrane devices. One of the advantages of the silicon / silica fabrication process presented in this paper is that it allows for a design in which both the microfluidics and nanopillar concentrators are fabricated simultaneously and in-plane. The large number of nanogaps that can be fabricated in parallel in one flow channel, coupled with a short channel length (1 μm) provide sufficient volumetric flow to allow for this simple design. Taken together, the nanogaps act as a silicon/silica-based membrane in-line with the microchannel flow. The only comparably simple design previously reported requires a Nafion-based preconcentrator [40]. Compared to polymer membrane devices, the presented fabrication process is more compatible with conventional lithography tools and cleanroom processes, making it amenable to scale-up to high volumes [5,33–37].

Furthermore, a combined pre-concentrator / filter design allows for bead-based immunoassays, as the micropillars both induce ICP and stop functionalized beads in the ICP enrichment zone. In contrast, most previously reported designs use separate features to perform enrichment and capture beads. For example, in Wang et al. [5], an additional shallower channel was fabricated to stop microbeads in front of the silica-based horizontal nanochannels; this requires additional mask alignment and etching steps. Likewise, in Ko et al. [39], additional sets of pillars were fabricated into their device to capture beads in front of Nafion membranes.

In our device, the coupling of ICP and electroosmotic flow (EOF) results in the accumulation of biomolecules in front of the pillars (detailed schematic diagram of mechanism in S1 Fig). In effect, the array of nanogapped pillars mimics the function of a semi-permselective membrane that prefers to transport cations rather than anions, given that the silica surface is negatively charged in phosphate buffered solution (PBS) at pH = 7.4. The permselectivity of silica nanogaps originates from surface conduction through the electrical double layer (EDL), which in microchannels is usually overwhelmed by bulk conduction. However, surface conduction becomes significant when the channel size is comparable to the thickness of the EDL. For example, if one were to fabricate nanoscale channels, the ratio of EDL thickness to channel width would increase by three orders of magnitude when compared to the effect in a microscale channel. It follows that the ratio of surface conductivity to bulk conductivity would also increase by three orders of magnitude (Dukhin number [46]).

Note that our nanogaps do not perform as a perfect ion selective membrane. The thickness of the EDL with 0.1X and 1X PBS is approximately 3 nm and 0.8 nm [28,37], respectively, both of which are significantly smaller than the size of our nanogaps (30 nm). There are two relevant conduction phenomenon in our nanogaps: *bulk* conduction—transporting both cations and anions and *surface* conduction—transporting mostly cations through EDL. At this scale (channel width ~10x larger than EDL thickness), the cations transported by surface conduction initiate a weak concentration polarization and decrease ion concentration on the anodic side of the nanostructure [27,28]. The lower ion concentration near the nanogaps increases the thickness of local EDL and enhances permselectivity which contributes to faster concentration polarization. Thus, the concentration polarization in our device depends on a positive feedback process [28] which develops strong ICP even at moderate (0.1X PBS, ~15 mM) ionic strength (i.e. buffers for which the Debye length is significantly shorter than the nanogap width). The low ion concentration region (depletion zone) induces nonequilibrium space charge layers, resulting in accelerated nonequilibrium EOFs in the vicinity of the nanostructures [37,46,47]. Due to the incompressibility of the fluid, strong vortical flows are generated in the depletion zone, driving the fluid back to the upstream [27,28,48,49]. At the same time, the low ion concentration amplifies the electric field in the depletion zone, repelling negatively charged biomolecules away from the nanostructures. The backflow of the vortices along with the strong electric field work jointly to move biomolecules upstream until the convective and electromigratory velocity balance, resulting in the continuous accumulation of biomolecules (Fig 1H) [48,50].

To demonstrate the bead immunoassay possibilities of our device, we chose interleukin 6 (IL6). IL6 is a proinflammatory cytokine and serves as an important mediator during the acute phase response to inflammation in sepsis. Its concentration in blood is used as a diagnostic for sepsis [51,52] and is significantly positively correlated with septic patient mortality rate [53]. As a life-threatening disease caused by systemic immune response, septic patients usually need immediate treatment. The ability to monitor IL6 levels time scales in the tens of minutes would open the door to closed loop, patient-specific sepsis management[54–56].

## Device fabrication

The preconcentration device is formed from two parallel channels connected by microchannels which contain the nano-gapped pillars; four reservoirs provide tubing and electrode connections (Fig 1A). During enrichment, one channel (left) is connected to the anode while the other channel (right) is connected to the cathode. As shown in Fig 1B–1D, the microchannels are 700 μm long, 30 μm wide and 6.5 μm deep and contain 397 micropillars. Each micropillar has a cross section of approximately 1 μm by 1 μm and the same height as the microchannels. The gaps between micropillars are 30 nm wide, forming 398 nanogaps.

All structures are fabricated in the silicon substrate. We chose silicon micromachining instead of PDMS molding because a high aspect ratio is achievable when etching silicon. The finished devices have three layers as shown in Fig 1E and 1F. The fabricated silicon device is sealed with a silica wafer spin coated with polysilsesquioxane (PSQ) and this transparent side enables fluorescence detection. A thick layer of PDMS with four through-holes is bonded on the other side of the silicon layer to form the reservoirs for tube and electrode connection (Fig 1G).

**Fig 2:**
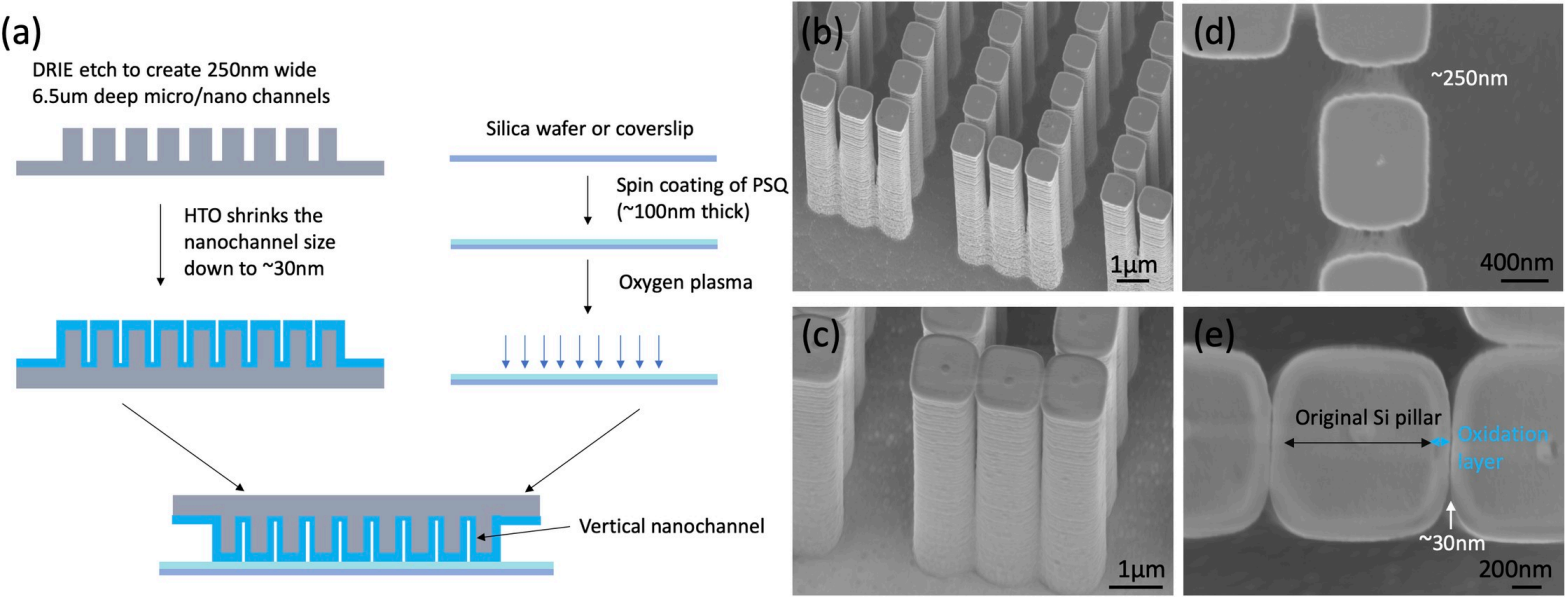
Fabrication of the nanofluidic device. (a) Schematic cross-section of fabrication process at the red line shown in Fig 1C. All channels and pillars were 6.5 μm tall and etched in one single DRIE step. (b)&(d) SEM of micropillars before HTO. (c)&(e) SEM of micropillars after HTO. The additional silicon dioxide layer increased the pillar size and reduced the nanogap size.

Microchannels (including micropillars) were fabricated on a p-type prime silicon wafer (Fig 2A). A 420nm thick layer of deep ultraviolet (DUV) photoresist (UV210-0.6, Dow Chemical, Midland, MI) was spun on a silicon wafer and then exposed in ASML 5500/300 (ASML, Veldhoven, Netherlands). The exposed photoresist was then developed in Megaposit MF26A (Dow Chemical, Midland, MI) and hard baked for 2 hrs at 120°C. The silicon wafer was etched 6.5 μm deep with deep reactive-ion etching (DRIE) in an ICP (inductively coupled plasma) etcher (SPTS Technologies, Newport, UK). Ideally, deeper nanogaps would give better results, but we were constrained to 6.5μm depths by the aspect ratio limits of the etch recipe. After spin coating a 10 μm thick layer of photoresist (SPR 220–7.0, Dow Chemical, Midland, MI) to protect surface structures, reservoirs were ground manually with a dental drill to create access holes through the silicon. This step could be replaced by RIE or KOH etching for scaled up manufacturing [5,33]. Because the minimum lithographically-defined linewidth was 250 nm (Fig 2B and 2D), the next step was to shrink the nanogaps from 250 nm to ~30 nm by oxide deposition. Since we had high aspect ratio structures, a highly conformal deposition process was used to achieve uniform deposition over the micropillars. We deposited a layer of high-temperature oxide (HTO) at 900° C over the etched devices with a TYTAN Diffusion Furnace System (Tystar, Garden Grove, CA) to achieve ~30 nm wide nanogaps (Fig 2C and 2E). Note, it is important to deposit oxide after reservoir grinding. Otherwise, the newly grinded reservoirs would expose the conductive silicon substrate inside the reservoirs and create an electrical short circuit when wet.

After the fabrication of all features on the silicon wafer, a PSQ (Gelest, Morrisville, PA) coated silica wafer (500 μm thick) was used to seal the channels. PSQ has a similar chemistry to PDMS, able to form covalent bonds with a silicon dioxide surface after plasma treatment and has been used for silicon/silica based device sealing previously [24,57,58]. PSQ was chosen over PDMS for our device because commercially-available PSQ has higher strength and is more amenable to dilution and spin coating. We used a PSQ sealing process adapted from that reported by Gu et al [58]. PSQ was diluted in xylene in a 3:2 ratio and then spin-coated on an oxygen plasma cleaned silica wafer at 3000 RPM for 1 min. After spin-coating, the silica wafer was baked overnight at 150°C. The hard-baked silica wafer and processed silicon wafer were then O_2_ plasma treated at 100 W for 60 seconds in a YES-G500 Plasma Cleaning System (Yield Engineering Systems, Livermore, CA). The plasma power and time were crucial to properly activate the surface and form covalent bonds between two surfaces. We found that high-energy plasma damaged the structure of the PSQ and weakened wafer bonding. Similarly, long duration plasma exposure likely reduced the number of effective silanol groups via back-biting scission reactions. These two effects are well-known in PDMS plasma bonding processes [59]. This bonding process can be replaced by anodic bonding for scale-up [5,33]; at lab scale, PSQ mediated bonding is easier and faster. After plasma treatment, the two surfaces were brought in contact immediately. After wafer bonding, the wafer was diced into 1 cm by 1 cm chips as seen in Fig 1E, and then a thick layer of PDMS with 4 through-holes was bonded to the backside of the chip after a backside O_2_ plasma treatment. This layer enabled connections between the tubing and the reservoirs.

## Materials and methods

For characterization experiments, Strep-647(Alexa-Fluor 647 conjugated streptavidin, Thermal Fisher Scientific, Waltham, MA) was used as the sample protein for preconcentration characterization in PBS (0.1X and 1X PBS, pH = 7.4) solution. We also added 0.1% Tween-20 (Thermo Fisher Scientific, Waltham, MA) in all solutions in this paper to reduce non-specific binding on side walls. In the experiments that varied voltage (25V, 12.5V, 5V) and strep-647 concentration (200ng/ml, 20ng/ml, 2ng/ml), we used 0.1X PBS (pH = 7.4) with 0.1% Tween-20.

For experiments employing fluorescent IL6, recombinant mouse IL6 (Thermo Fisher Scientific, Waltham, MA) was labeled with Alexa-Fluor 488 using the Microscale Protein Labeling Kit (Thermo Fisher Scientific, Waltham, MA). All labeled IL6 (IL6-488) preconcentration experiments were performed in 0.1X PBS (pH = 7.4,7.8,9) with 0.1% Tween-20 at 25V. Sodium hydroxide (NaOH) were used to adjust the pH of 0.1X PBS solution. Both labeled and non-labeled IL6 were diluted in 0.1X PBS (pH = 7.8) with 0.1% Tween-20 for bead-based immunoassays, while a washing buffer was created with 1X PBS with 0.1% Tween-20. Strep-647 and detection antibody (BD Bioscience, San Jose, CA) were diluted in the washing buffer at the concentration of 2 μg/ml and 1 μg/ml, respectively, in immunoassays for non-labeled IL6. BSA-488 (Alexa Fluor 488 conjugated bovine serum albumin, Thermo Fisher Scientific, Waltham, MA) and BSA-647 (Alexa Fluor 647 conjugated bovine serum albumin, Thermo Fisher Scientific, Waltham, MA) were used as tracers for non-labeled IL6 and labeled IL6, respectively.

Carboxyl group-coated polystyrene microbeads (~4.6 μm diameter, Spherotech,Lake Forest, IL) were conjugated with capture antibody of mouse IL6 by conventional EDC (1-Ethyl-3-(3-dimethylaminopropyl)-carbodiimide, Thermo Fisher Scientific, Waltham, MA) and Sulfo-NHS (N-hydroxysulfoxuccinimide, Thermo Fisher Scientific, Waltham, MA) chemistry. Briefly, EDC and Sulfo-NHS were first dissolved in 0.05M MES buffer (Thermo Fisher Scientific, Waltham, MA) and immediately added to 0.5% w/v microbeads solution in DI water. The mixed solution was incubated for 15 min at room temperature, which was followed by centrifugation and wash in DI water with 0.1% Tween 20 in order to remove excessive EDC and Sulfo-NHS. Tween-20 prevented microbeads from aggregating and adhering to centrifuge tube surfaces during centrifugation. Because Sulfo-NHS hydrolyzes in water spontaneously, the washing process had to be fast. After washing at least 3 times, anti-IL6 capture antibody (BD Biosciences, San Jose, CA) was added into the microbead solution and incubated for 2 hrs at room temperature. Then, quencher solution, 50% w/v hydroxyl amine (Sigma-Aldrich, St. Louis, MO), was added into the mixture and incubated for another 10 min to deactivate the remaining NHS, after which the microbeads were washed again in DI water with 0.1% Tween-20. Before using the antibody conjugated microbeads, we blocked the microbeads by incubating in 1% BSA (bovine serum albumin, Thermo Fisher Scientific, Waltham, MA) for 1 hr at room temperature.

All fluorescent images were taken on an inverted fluorescent microscope (Nikon Eclipse Ti, Nikon Instruments Inc, Melville, NY) with a CCD camera such that the anode was on the left side and the cathode on the right side of the image. Ag/AgCl electrodes (A-M System, Sequim, WA) were used to apply voltage to each reservoir. A waveform generator (Keysight,Santa Rosa, CA) connected to an amplifier (Tegam, Geneva, OH) was used to apply DC Voltage. ImageJ was used for image analysis.

## Results

### Lower ionic strength results in stronger enrichment

**Fig 3:**
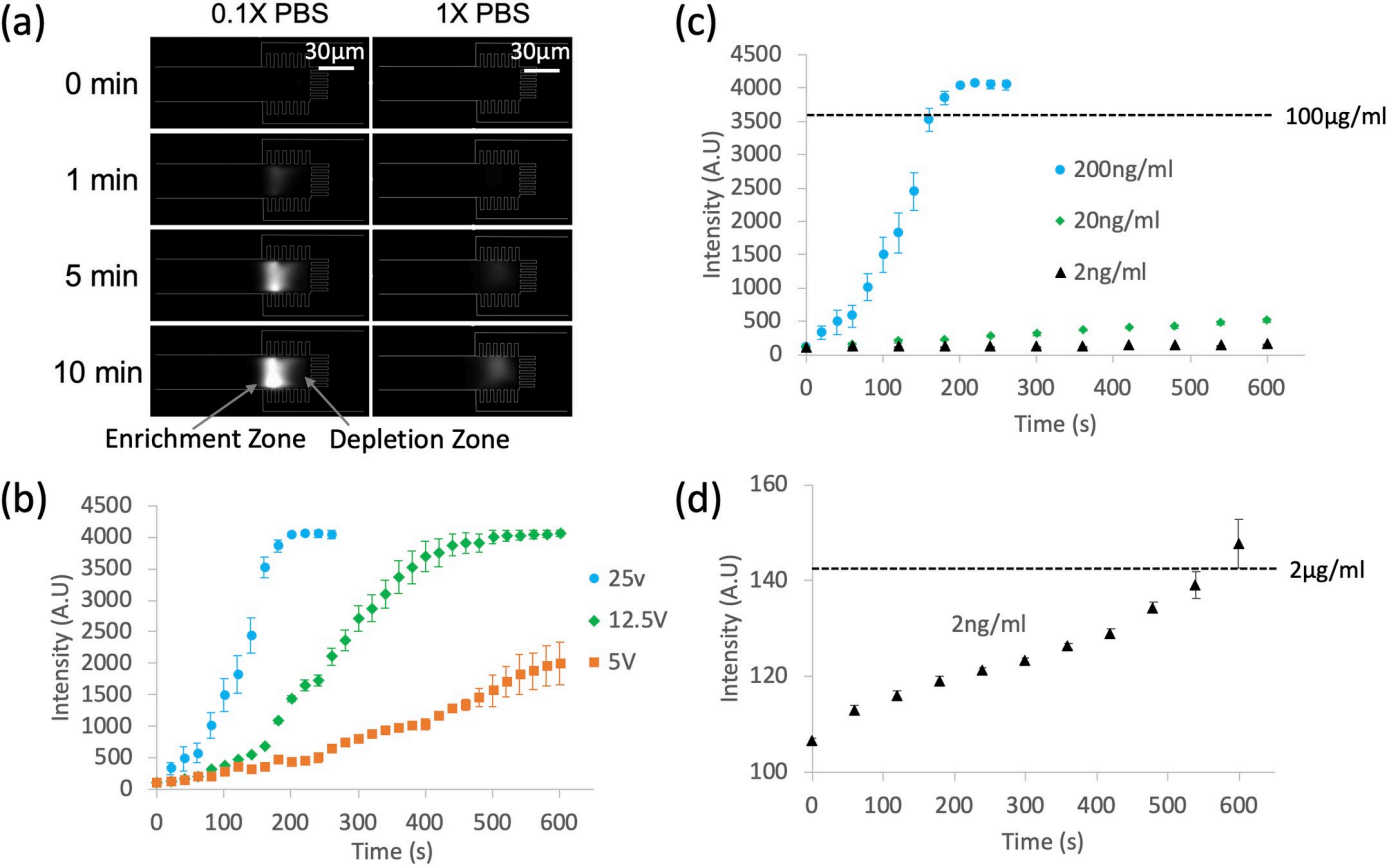
Characterization of protein enrichment. (a) Protein (200 ng/ml Strep-647) enrichment in different ionic strengths (0.1X & 1X PBS with 0.1% Tween-20). ICP based protein enrichment is much more effective in 0.1X PBS than in 1X PBS. A DC voltage of 12.5V was applied across the channels with anode on the left side and cathode on the right side. (b) Enrichment speed as a function of the applied DC voltage (5 V, 12.5 V and 25 V). Higher voltage leads to faster enrichment of 200 ng/ml Strep-647 solution. (c) Enrichment over 10min with various Strep-647 concentrations at 25V. (d) Closeup view for the 2ng/ml Strep-647 experiment in (c). Note the ~1000 fold enrichment in 10min. All sample solutions in (b-d) were diluted in 0.1X PBS with 0.1% Tween-20.

We characterized the preconcentration phenomenon under different conditions in order to find the optimal ionic strength, voltage, and pH. Fig 3A shows the protein enrichment behavior under different ionic strengths of buffer solution (0.1X and 1X PBS). Strep-647 at a concentration of 200 ng/ml was used as the fluorescent tracer. When applying a 12.5 V DC voltage, we could observe the formation of an enrichment zone on the anodic (left) side of nanogaps. As soon as no voltage applied, accumulated strep-647 molecules easily leak through the nanogaps with positive pressure from anodic side (S1 Video). Note that the streptavidin molecules (~5 nm diameter [60]) are much smaller than the nanogaps: enrichment is caused by the development of ICP instead of size filtering. As shown in Fig 3A, detectable accumulation of strep-467 occurred after 1 min in 0.1X PBS, but was not detectable until 5 min in 1X PBS. After 10min, the intensity saturated in 0.1X PBS, while the intensity in 1X PBS was only approximately 30% of the saturated level.

### Higher voltage leads to faster enrichment

We also investigated the preconcentration effect as a function of the applied DC voltage. Fig 3B shows that higher voltage leads to faster preconcentration. It took 600 s to achieve a 2000 A.U intensity at 5 V, while it took approximately 240–260 s and 120 s at 12.5 V and 25 V, respectively, to reach the same intensity. In this voltage range, the enrichment speed is roughly proportional to the voltage, which is in agreement with the theoretical relationship between EOF and applied voltage.

### The preconcentrator achieves 1000-fold enrichment

After determining the optimal ionic strength and voltage, we characterized the enrichment phenomenon with various concentrations of strep-647. Fig 3C and 3D shows the preconcentration performance for strep-647 at 25 V in 0.1X PBS (pH = 7.4) with 0.1% Tween-20. Fig 3C shows the enrichment of strep-647 at initial concentrations of 200ng/ml, 20 ng/ml, and 2 ng/ml over 10 min. During the preconcentration, one fluorescent image was taken every minute. Between image captures, the shutter for the excitation light was closed to prevent photobleaching. Fig 3D is a closer look at the 2ng/ml group in Fig 3C. The horizontal dashed line is the signal intensity of a 2 μg/ml strep-647 solution, provided for reference. During protein concentration, the fluorescent intensity of an initially 2 ng/ml solution increased, exceeding the 2 μg/ml reference level within 10 min, a 1000-fold enrichment.

### IL6 can be enriched with pH adjustment

**Fig 4:**
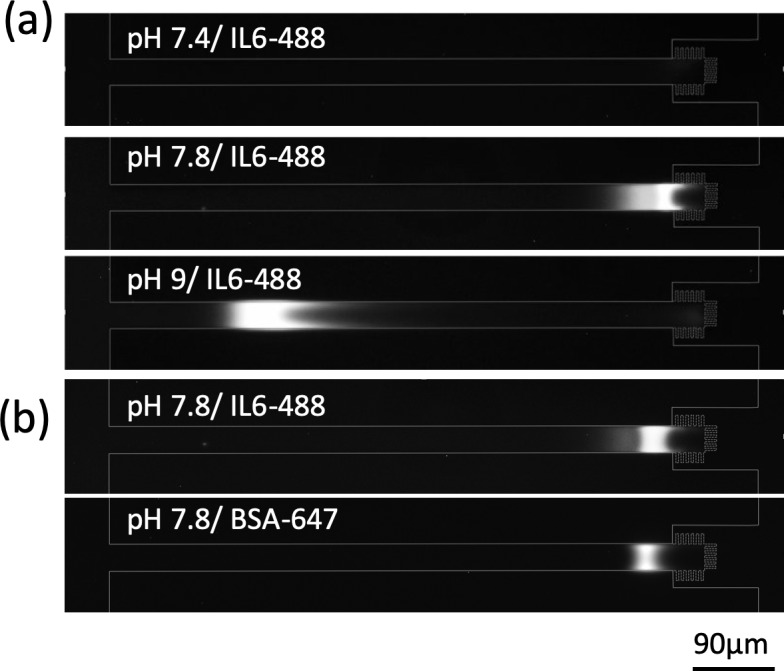
BSA is a good tracer for IL6 preconcentration. (a) The enrichment performance of IL6-488 under various pH. Sample solution (0.1X PBS, 0.1% Tween-20, 2μg/ml IL6-488 at pH = 7.4, 7.8, 9) was loaded into the device and 25V was applied to the reservoirs for 10min. (b) BSA-647 could be used as a good tracer for IL6 enrichment. Both BSA-647 (200 ng/ml) and IL6-488 (2 μg/ml) were enriched simultaneously at 25V in the same sample solution as (a) except the pH = 7.8. The enrichment zone of BSA-647 was highly colocalized with the enrichment zone of IL6-488. To maintain the activity of IL6, we used a low degree of labeling for IL6-488 (less fluorescent molecules per protein molecule). Thus, we used 10 times higher concentration of IL6-488 than BSA-647 for characterization experiments.

Before integrating immunoassays into our device, we used IL6-488 to demonstrate that the preconcentrator could enrich not only streptavidin but also IL6. However, there was no enrichment of IL6-488 in 0.1X PBS at pH = 7.4, as shown in Fig 4A. The IL6-488 enrichment was then tested at higher pH (pH = 7.8, 9). Because the IL6 molecules are more negatively charged at higher pH, we expected them to accumulate more easily within the depletion zone(Fig 4A). Note that the antigen-antibody reaction is negatively affected by higher pH (pH > 8), which reduces the affinity of antibodies [61]. We tested the antibody activity by incubating IL6 with antibody-conjugated beads at various pH(pH = 7.4, 7.8, 9). For the same concentration of IL6, the signal intensity on beads was at least 60% lower at pH = 9 compared to that at pH = 7.4 due to the lower affinity of the antibody at higher pH(S2 Fig). Thus, for all of the following immunoassays, we chose the lowest pH (pH = 7.8) that promoted enrichment but did not reduce intensity below about 90% of the intensity observed at pH 7.4.

### BSA-647 is a good fluorescent tracer for IL6 preconcentration

In order to monitor the development of ICP and an enrichment zone in the IL6 immunoassay, we used a fluorescent tracer for IL6. We required a fluorescent tracer that 1) enriched along with IL6, 2) accumulated at the same location as IL6, and 3) did not interfere with other reagents in the immunoassay. As shown in Fig 4B, when enriched simultaneously along with IL6-488 in the same solution, BSA-647 enrichment zone was colocalized with the enrichment zone of IL6-488. Therefore, we used BSA-647 as our fluorescent tracer for IL6 immunoassays at pH = 7.8.

### Nanogap preconcentrator enables bead-based immunoassays

To demonstrate the compatibility of this method with bead-based immunoassays, we conjugated polystyrene microbeads (4.6 μm in diameter) with rat anti-mouse IL6 capture antibody. Since the microbeads were smaller than the height of the microchannels but much larger than the nanogaps, all microbeads were stopped at the pillars. Smaller beads (100 nm– 2 μm in diameter, data not shown) also stopped at the pillars, but we chose ~4.6 μm diameter beads because the larger size provided better observation and control over the number of beads per microchannel. In addition, beads with submicron size could potentially partially block the nanogaps and decrease the effective nanogap size, which would significantly affect enrichment behavior.

**Fig 5:**
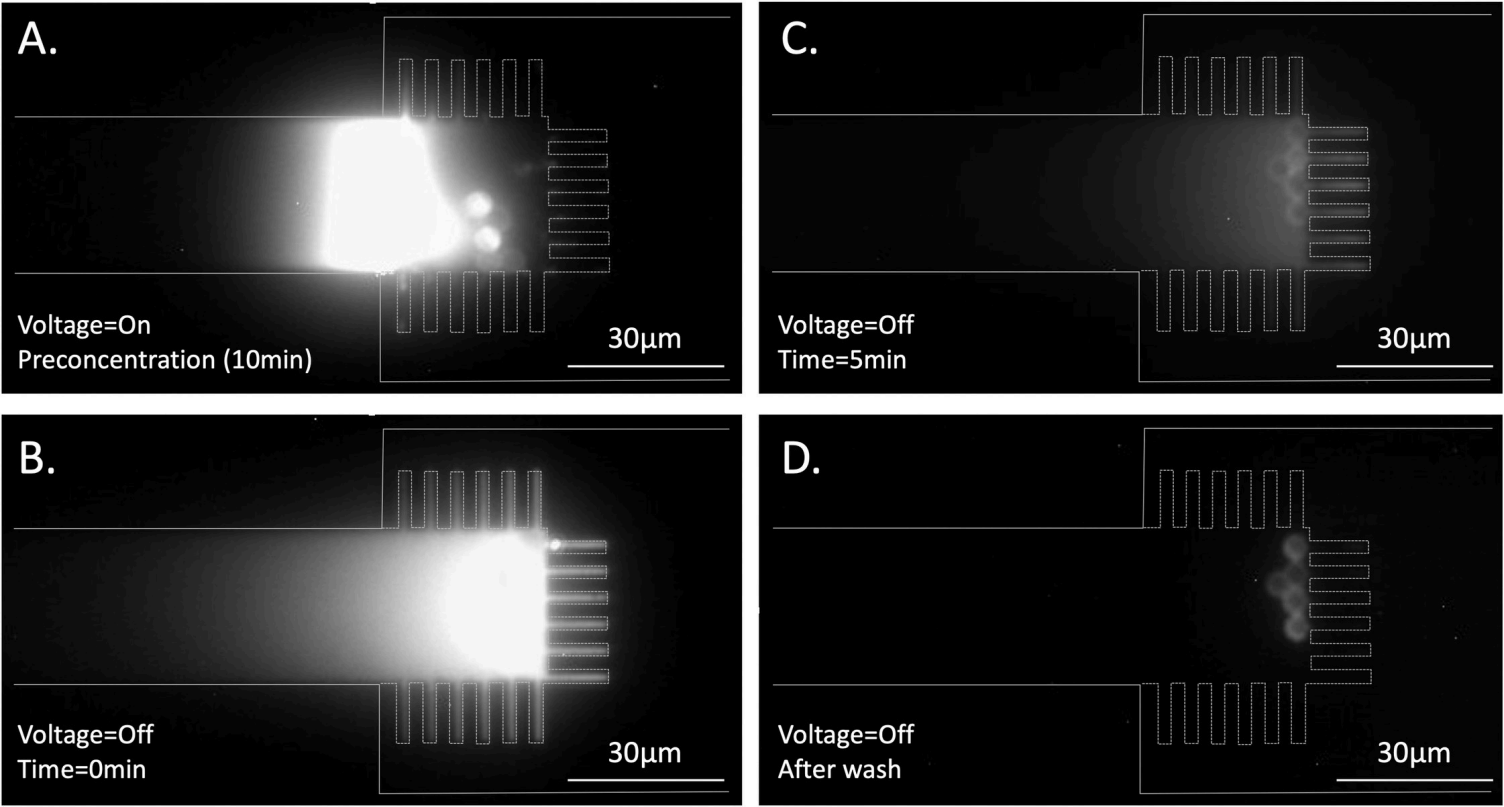
Immunoassay procedures for IL6-488. (a) Bead loading (~5 microbeads per microchannel) and preconcentration of IL6-488 at 25V for 10 min in sample solution (0.1X PBS, 1% Tween-20, 500 ng/ml IL6-488, pH = 7.8). Immediately after turning off the voltage (b), the enrichment molecules started diffusing away toward both sides. Since the nanogaps restricted diffusion, the diffusion to right side was slower. Thus, even 5 min after turning off the voltage, a higher fluorescent signal was detectable in front of the nanostructure (c). (d) After 5min incubation, a positive pressure was applied through anodic side reservoirs to wash away excessive IL6-488.

The immunoassay process is shown in Fig 5. First, approximately five microbeads were loaded into each microchannel, followed by the loading of IL6-488 sample solution. DC voltage was applied for 10 min at 25 V. Note, similar to other ICP based preconcentrators[28,33,35,37,39,40,42,62], we also observed vortical flow in the depletion region so that microbeads moved along with the vortical flow during enrichment (S2 Video). The location of microbeads in Fig 5C and 5D is, therefore, different from the location in Fig 5A. The microbeads were incubated for five minutes after turning off the voltage (Fig 5B and 5C). Since the nanogaps restricted diffusion, IL6-488 diffusion was slower in the nanostructures than in an open microchannel (where molecules can diffuse freely in both directions). Thus, after a 5 min incubation, the elevated fluorescent signal (Fig 5C) was ~75% lower than the signal immediately after turning off the voltage (Fig 5B), but it was still more than 4x higher than the background. This result demonstrated that without additional convection, IL6 molecules did not diffuse away immediately. This additional 5 min incubation improved antigen-antibody binding and noticeably enhanced signal intensity [40], after which a positive pressure was applied through anodic side to wash away excessive fluorescent molecules (Fig 5D).

**Fig 6:**
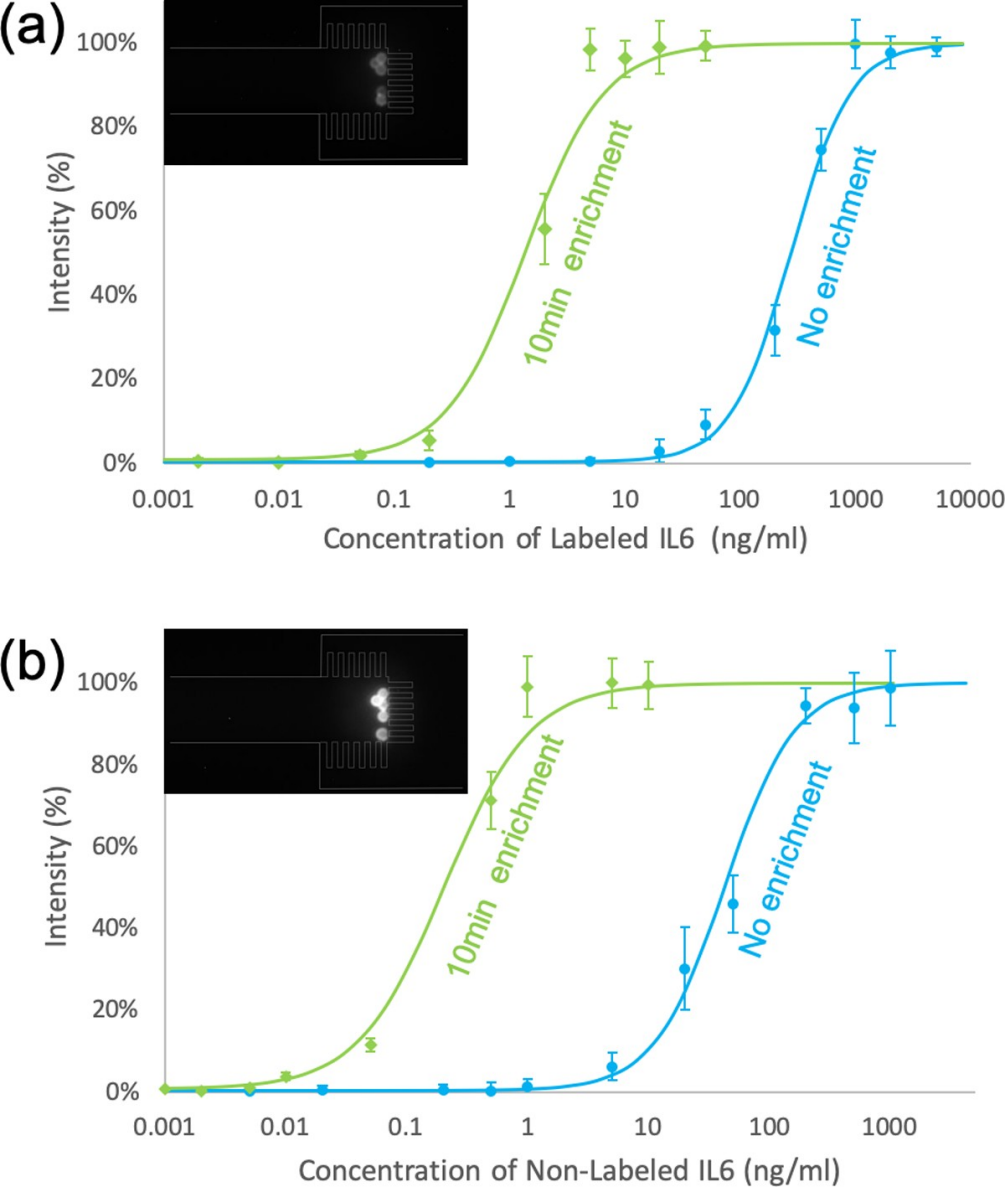
Dose response of the immunoassay with and without preconcentration. Immunoassays for (a) IL6-488 and (b) non-labeled IL6 were performed on beads with (green curve) and without (blue curve) enrichment. The sensitivity of IL6-488 and non-labeled IL6 detection was enhanced ~125 fold and ~162 fold, respectively. The in-plot fluorescence images show saturated signals for IL6-488 (a) and non-labeled IL6 (b). Both labeled and non-labeled IL6 preconcentration was conducted with the same sample solution (0.1X PBS, 1% Tween-20, pH = 7.8), DC voltage (25 V) and duration (10 min). BSA-647 or BSA-488 (200 ng/ml) was used as the tracer protein and simulated molecular background for labeled and non-labeled IL6, respectively. Both of the non-enrichment groups were incubated in tubes for 2 hrs at room temperature in the same sample solution.

The detection of non-labeled IL6 uses the same process as IL6-488 detection, except that biotin conjugated detection antibody and strep-647 were loaded after loading IL6. An additional washing step was necessary between the loading of detection antibody and strep-647, since the mixture of these two reagents might induce micro-aggregation and block the nanogaps. The dose response curves of both labeled and non-labeled IL6 are shown in Fig 6. Two inset fluorescence images show the saturated signals for each case. Fluorescent signals were normalized using the saturated signal and zero dose signal as 100% and 0% intensity. We used the four parameter logistic model in Eq 1 to fit the dose response curves which models the physicochemical mechanism of antigen-antibody binding

$$\text{y}=\text{d}+\frac{a-d}{1+{\left(\frac{x}{c}\right)}^{b}}$$ Eq 1

where *y* is the fluorescent intensity, *x* is the target concentration, *d* represents the intensity at infinite concentration, *a* is the estimated intensity at zero concentration of target, *b* is the Hill coefficient referring to the steepness of the curve, and *c* is the midrange concentration [39,63]. All fitted curves had *R*^*2*^ (coefficient of determination) values larger than 0.98. Three standard deviations above the background signal was used to define the limit of detection (LOD). Using these curves, the LOD of labeled and non-labeled IL6 without enrichment was 15ng/ml and 1.2ng/ml, respectively. After 10 min enrichment, the LOD of labeled and non-labeled IL6 was enhanced to 120 pg/ml (~125 fold improvement) and 7.4pg/ml (~162 fold improvement), which is comparable to the 500 fold enhancement in 60 min (30 min enrichment plus 30 min additional incubation) from the previously reported straight PDMS/Nafion microchannel design [40].

## Discussion

The weaker enrichment observed for 1X PBS vs. 0.1X PBS (Fig 3A) is likely due to the higher ionic strength of the solution. A higher ionic strength solution would exhibit a thinner EDL and, therefore, slower electroosmotic flow (assuming the same DC voltage applied) and a smaller ratio of surface conductivity to bulk conductivity. Thus, it takes longer to develop a low concentration region near the nanogaps and accumulate target molecules. Interestingly, no fluorescent signal was found for 0.01X PBS. One possible reason is that this much lower ion concentration promotes strong ICP immediately and a depletion zone develops which is longer than the microchannel, preventing protein enrichment.

Also of interest is that for voltages of 5–25V, the enrichment rate of our preconcentrator is proportional to the voltage, because higher electric potential results in higher electroosmotic flow rate. Electroosmotic flow is the principal effect bringing proteins to the enrichment zone from bulk areas. Thus, a higher electroosmotic flow rate accelerates the protein accumulation process. However, if the voltage is too low (< 2V), the depletion zone did not fully develop and was not effective at preventing analytes from moving downstream [50]. When the voltage was higher than 50 V, the EOF overwhelmed the ICP-based enrichment so the enrichment zone became unstable. Consequently, strep-647 molecules leaked through nanogaps frequently. In addition, when the voltage was very high (>100 V), background proteins (such as the BSA used in the immunoassays in section 3.6) started to aggregate and to block the nanogaps (S3 Fig). This may be due to pH changes [64] or ohmic heating [65–68] but further investigation is needed to be certain.

Unlike Strep-647 (pI ~5.5 [69]), IL6-488 (pI ~6.96 [70]) could not be enriched at pH 7.4. We believe this is because the pI value of mouse IL6 is very close to the pH of PBS (pH = 7.4); thus, mouse IL6 molecules are either not charged or weakly charged in PBS. According to the mechanism of ICP-based enrichment, neutral molecules cannot be trapped efficiently in front of the depletion zone [28]. In contrast, IL6-488 was successfully enriched at higher pH (pH = 7.8, 9). There are two possible reasons. First, more silanol groups on silica surface are deprotonated in higher pH [71]. The higher charge density on nanogap surfaces leads to higher surface conductivity, which enhances ICP development. Second, the higher pH also leads to more negatively charged IL6-488 molecules, so that the depletion zone can trap IL6-488 molecules more efficiently, preventing them moving downstream [27]. At pH = 9, the enrichment zone was approximately 450μm away from the nanostructure (Fig 4A), which was only 36μm at pH = 7.8. This is likely due to higher surface charge density producing stronger ICP development and the depletion zone propagating further at pH = 9. In addition, increasing pH increases the electrophoretic mobility of protein molecules (i.e. more negatively charged at higher pH), so that the vortical flows carry these molecules further back to the location of lower electric field, where the convection and electromigration balance [29,32].

Lastly, the saturated signal for non-labeled IL6 (Fig 6B inset image) was much higher than the saturated signal for labeled IL6 (Fig 6A inset image). There are three possible reasons for this. First, since each detection antibody molecule has multiple biotin sites and each strep-647 molecule has multiple fluorophores, this combination amplifies the signal. Second, we used a lower degree of labeling for IL6 so there were fewer fluorophores per IL6 molecule. Third, the labeled IL6 and non-labeled IL6 used different fluorescent molecules for detection. The Alexa Fluor 647 on Strep-647 (for non-labeled IL6) is generally brighter than the Alexa Fluor 488 on IL6-488.

Although we successfully enriched IL6 and enhanced the sensitivity by more than 2 orders of magnitude, the sensitivity enhancement factor was still lower than the concentration factor, which was at least 1000 times greater than initial concentration. This might be result of the local pH change, ohmic heating and the change in buffer ionic strength during preconcentration [40]. However, more investigation is needed to identify which factor plays the main role.

## Conclusion

In summary, we demonstrated the use of nanogaps fabricated between micropillars to mimic a semi-permselective membrane for the purpose of ICP-based target molecule preconcentration. Since the nanogaps have a high ratio of surface conductivity to bulk conductivity, ICP based protein preconcentration was generated on the anodic side of the micropillars. The large number of short vertical nanogaps allowed sufficient volumetric flow for bead loading and washing, which is not possible for conventional horizontal nanochannels. This structure is comparable in performance to preconcentration microchannel designs using Nafion film [40] but is more amenable to large scale fabrication. We also demonstrated a preconcentration-enhanced immunoassay for the detection of IL6. This structure and preconcentration mechanism can be useful to improve the efficiency and the multiplexing capability of silicon/silica based preconcentrators. The performance of this device can be tuned by increasing the number or changing the dimensions of the pillars without changing the fabrication process. We expect this assay can be used for a broad range of analytes and a variety of detection methods. For example, this device can be combined with faster label-free detection methods to further speed up assays and eliminate the additional fluid control needed for bead loading and washing.

## Supporting information

S1 Fig The mechanism of ICP and biomolecule enrichment in front of nanostructure with the anode on the left side of the nanostructure. (a)The negatively charged nanogaps transport more cations than anions. (b)The additional cations transported by surface conduction initiate a weak concentration polarization and decreases ion concentration on the anodic side of the nanogaps. The lower ionic strength increases the thickness of local EDL, which enhances permselectivity of nanogaps. This positive feedback promotes a strong concentration polarization even when the Debye length is significantly shorter than the nanogap width. (c) The depletion zone induces nonequilibrium space charge layers and generates nonequilibrium EOFs near nanogaps. Since the fluid is incompressible, strong vortical flows are generated in the depletion zone. The backwards fluid flows along with the amplified electric field in depletion zone move analytes (negatively charged) upstream until the convection and electromigration balance, resulting in analyte enrichment.
(TIF)

S2 Fig Higher pH negatively affects antibody-antigen reaction. In this experiment, microbeads were incubated with 200 ng/ml non-labeled IL6 for 2 hr, followed by the incubation in detection antibody and strep-647, respectively. The IL6 solutions were prepared at different pH. The intensity of microbeads at pH = 9 is more than 60% lower than that at pH = 7.4.
(TIF)

S3 Fig Protein aggregation induced by high voltage. This experiment was conducted with the same immunoassay process described in the paper, except that the DC voltage used for enrichment was 100 V. Yellow arrow point at beads and white arrows indicate the protein aggregates. Nanostructure should be non-fluorescent as seen in Fig 3–6. We can see the nanostructure in this image because the nanogaps were completely blocked by a layer of protein aggregates, indicated by white dashed arrows.
(TIF)

S1 Video As soon as turning off the applied DC voltage (25V), accumulated strep-647 molecules leaked through nanogaps with positive pressure from anodic side. Thus, the protein molecule accumulation was done by ICP instead of size filtering. The volumetric flow through nanostructure was relatively fast, which enabled bead loading and washing steps to integrate immunoassays into our device. The video plays in real time.
(MP4)

S2 Video There were two vortical flow in depletion region, which was visualized in video with the movement of microbeads. BSA-488 was used as the fluorescent tracer for enrichment zone. The video plays in real time.
(MP4)
