# Correction: Ion concentration polarization (ICP) of proteins at silicon micropillar nanogaps

**DOI:** 10.1371/journal.pone.0229405

**Published:** 2020-02-13

**Authors:** Bochao Lu, Joe W. Chen, Michel M. Maharbiz

Joe W. Chen should be included in the author byline. Joe W. Chen should be listed as the second author, and his affiliation is 1: UC Berkeley-UCSF Graduate Program in Bioengineering, University of California–Berkeley, Berkeley, California, United States of America. The contributions of this author are as follows: Methodology. The correct citation is: Lu B, Chen JW, Maharbiz MM (2019) Ion concentration polarization (ICP) of proteins at silicon micropillar nanogaps. PLoS ONE 14(11): e0223732. https://doi.org/10.1371/journal.pone.0223732

The Data Availability statement is incorrect. The raw data underlying Figs 3 and 6 are not provided in the published paper and its Supporting Information files. The authors have provided the raw data via OSF: https://osf.io/za9xc.
