# Supplementary figures and images for: Ion concentration polarization (ICP) of proteins at silicon micropillar nanogaps
Source: PLoS One. 2019 Nov 4;14(11):e0223732. doi: 10.1371/journal.pone.0223732 (PMC6827887; doi:10.1371/journal.pone.0223732)

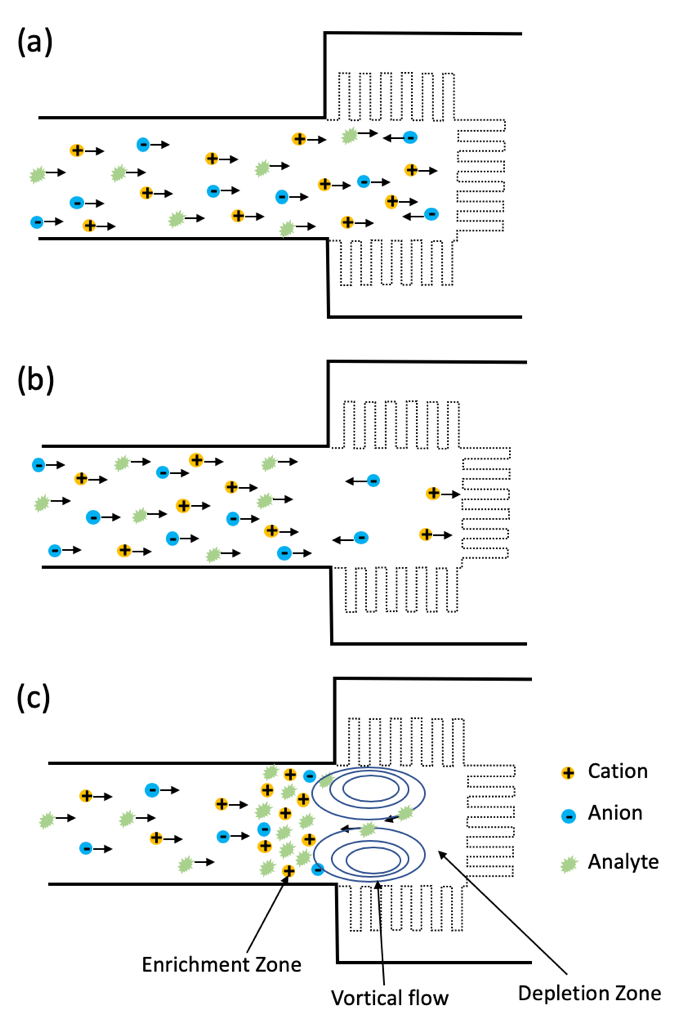

Supplement: S1 Fig — (a)The negatively charged nanogaps transport more cations than anions. (b)The additional cations transported by surface conduction initiate a weak concentration polarization and decreases ion concentration on the anodic side of the nanogaps. The lower ionic strength increases the thickness of local EDL, which enhances permselectivity of nanogaps. This positive feedback promotes a strong concentration polarization even when the Debye length is significantly shorter than the nanogap width. (c) The depletion zone induces nonequilibrium space charge layers and generates nonequilibrium EOFs near nanogaps. Since the fluid is incompressible, strong vortical flows are generated in the depletion zone. The backwards fluid flows along with the amplified electric field in depletion zone move analytes (negatively charged) upstream until the convection and electromigration balance, resulting in analyte enrichment. (TIF) [file pone.0223732.s001.tif]

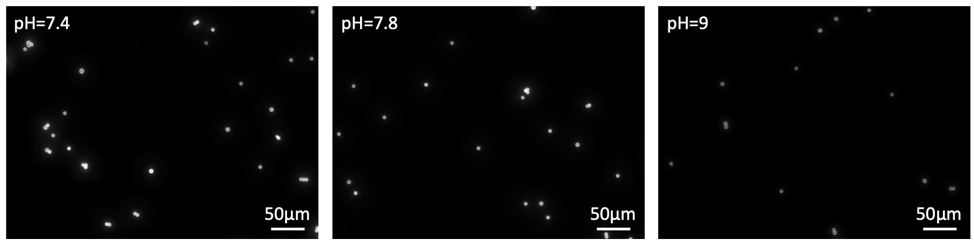

Supplement: S2 Fig — In this experiment, microbeads were incubated with 200 ng/ml non-labeled IL6 for 2 hr, followed by the incubation in detection antibody and strep-647, respectively. The IL6 solutions were prepared at different pH. The intensity of microbeads at pH = 9 is more than 60% lower than that at pH = 7.4. (TIF) [file pone.0223732.s002.tif]

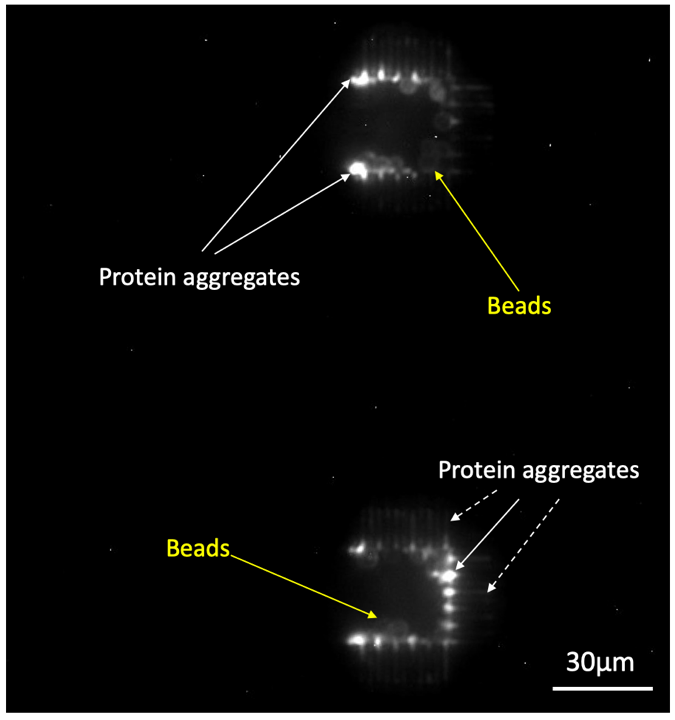

Supplement: S3 Fig — This experiment was conducted with the same immunoassay process described in the paper, except that the DC voltage used for enrichment was 100 V. Yellow arrow point at beads and white arrows indicate the protein aggregates. Nanostructure should be non-fluorescent as seen in Fig 3–6. We can see the nanostructure in this image because the nanogaps were completely blocked by a layer of protein aggregates, indicated by white dashed arrows. (TIF) [file pone.0223732.s003.tif]
